# Overall and cause-specific mortality in patients with dementia: a population-based cohort study in Taiwan

**DOI:** 10.4178/epih.e2023082

**Published:** 2023-08-31

**Authors:** Chia-Lun Kuo, Pei-Chen Lee, Li-Jung Elizabeth Ku, Yu Sun, Tsung-Hsueh Lu, Muhammad Atoillah Isfandiari, Chung-Yi Li

**Affiliations:** 1 Department of Public Health, National Cheng Kung University College of Medicine, Tainan, Taiwan; 2 Department of Psychiatry, Tsaotun Psychiatric Center, Ministry of Health and Welfare, Nantou, Taiwan; 3 Department of Neurology, En Chu Kong Hospital, New Taipei City, Taiwan; 4 Department of Neurology, National Taiwan University Hospital, National Taiwan University College of Medicine, Taipei, Taiwan; 5 Division of Epidemiology, Department of Epidemiology, Biostatistics and Demography, Health Promotion and Behavioral Science Faculty of Public Health, Universitas Airlangga, Surabaya, Indonesia; 6 Department of Public Health, China Medical University College of Public Health, Taichung, Taiwan; 7 Department of Healthcare Administration, Asia University College of Medical and Health Science, Taichung, Taiwan

**Keywords:** Causes of death, Death certificate, Dementia, Alzheimer dementia

## Abstract

**OBJECTIVES:**

OBJECTIVES: Information regarding the underlying causes of death (UCODs) and standardized mortality ratio (SMR) of dementia is instrumental in formulating medical strategies to prolong life in persons with dementia (PWD). We examined the leading UCODs among PWD and estimated the overall and cause-specific SMRs in relation to dementia in Taiwan.

**METHODS:**

Data were retrieved from 2 national datasets: the Taiwan Death Registry and the medical claim datasets of the National Health Insurance program. The observed person-years for each study participant were counted from the date of cohort enrollment to either the date of death or the final day of 2016. Sex-specific and age-specific SMRs were then calculated.

**RESULTS:**

The leading UCOD was circulatory disease, accounting for 26.0% of total deaths (n=3,505), followed by respiratory disease at 21.3% (n=2,875). PWD were at significantly increased risk of all-cause mortality (SMR, 2.01), with SMR decreasing with advancing age. A cause-specific analysis revealed that the highest SMRs were associated with nervous system diseases (SMR, 7.58) and mental, behavioral, and neurodevelopmental disorders (SMR, 4.80). Age appeared to modify SMR, suggesting that younger age at cohort enrollment was linked to higher SMRs for nearly all causes of mortality.

**CONCLUSIONS:**

Circulatory and respiratory diseases were the leading UCODs among PWD. The particularly elevated mortality due to nervous system diseases and mental disorders suggests that allocating more resources to neurological and psychiatric services is warranted. The elevated SMRs of various UCODs among younger PWD underscore the need for clinicians to pay particular attention to the medical care provided to these patients.

## GRAPHICAL ABSTRACT

[Fig f1-epih-45-e2023082]

## INTRODUCTION

Dementia is a neurodegenerative disease with multiple causes, characterized by a progressive decline in cognitive function and daily living activities. Previous studies have indicated that the prevalence of dementia has been on the rise globally in recent decades, with a particularly rapid increase in low-income and middle-income countries [1]. The global number of dementia cases rose from 20.2 million in 1990 to 43.8 million in 2016, primarily due to population aging and growth [2].

The heightened risk of mortality in people with dementia (PWD) has been frequently documented [3]. Dementia is associated with a high risk of mortality from a variety of chronic and acute causes, with the specific causes of death often being diverse. Diseases of the respiratory system, such as bronchopneumonia, and of the circulatory system, such as ischemic heart disease, are common causes of death among PWD [4,5]. Furthermore, research indicates that dementia amplifies the risks of acute organ dysfunction in hospitalized elderly patients [6]. Acute causes of death, such as accidents resulting from falls or agitated behavior, are also common among PWD. These individuals often experience behavioral and psychological symptoms of dementia throughout the course of the disease [7].

Although numerous studies have been conducted on the comorbidity and mortality risk factors associated with dementia, the discrepancies and heterogeneity in cause-of-death coding continue to pose challenges. Few recent nationwide population-based studies exist on the underlying causes of death (UCODs) in PWD, as well as the standardized mortality ratio (SMR) comparing the mortality risk between PWD and the general population. Taiwan is among the nations with rapidly aging populations. In 2018, Taiwan transitioned into an aged society, with the elderly population reaching 14% of the total. By the end of October 2022, the population aged over 65 years had grown to 4,042,790, accounting for 17.42% of the overall population [8]. Given this trend, the prevalence of dementia in Taiwan is anticipated to increase, as is the need for care. However, comprehensive documentation is lacking regarding the UCODs in PWD within this population. The aim of this study was to examine the distribution of UCODs among PWD in Taiwan, as well as to quantify the extent to which dementia may elevate mortality rates in PWD across various sex and age groups.

## MATERIALS AND METHODS

### Data sources

The data analyzed in this study were obtained from 2 national datasets overseen by the Health and Welfare Data Science Center (HWDSC) of the Ministry of Health and Welfare. These datasets included the Taiwan Death Registry (TDR) and the medical claim datasets from the National Health Insurance (NHI) program. The National Health Research Institute supplied a database of 2,000,000 individuals, who were randomly selected in 2005 and tracked until the end of 2016. No statistically significant differences were present in age, sex, or average insured payroll-related amount between the study sample and all NHI enrollees [9]. The NHI program in Taiwan is managed and supervised by the National Health Insurance Administration, which is part of the Ministry of Health and Welfare. This program provides universal medical insurance coverage for nearly all Taiwanese residents, accounting for more than 99% of the population [10].

In Taiwan, it is a legal requirement to register all live births and deaths within 10 days following the event. Death certificates provide a range of information, including the UCOD and various demographic variables. The TDR has been evaluated with respect to data quality and deemed valid [11].

### Study design

This retrospective cohort study included individuals who received a primary diagnosis of dementia between 2005 and 2014, and who had at least 2 outpatient medical claims or 1 hospital admission within a 1-year period. The enrollment date was determined by the first medical claim for dementia made between 2005 and 2014. The diagnosis was established based on the International Classification of Diseases, 9th revision, Clinical Modification codes indicating senile dementia (290.0), presenile dementia (290.1), Alzheimer disease (AD; 331.0), vascular dementia (290.4), and other types of dementia (331.1, 331.82, and 332), along with the International Classification of Diseases, 10th revision, Clinical Modification codes F03.9, G30, F01.5, G31.09, and G31.83.

The patients involved in the study were linked to the TDR using unique personal identification numbers. This method was employed to identify those patients who died during the study period, which spanned from 2005 to 2016.

### Statistical analysis

We began by detailing the characteristics of the study participants. We also provided the numbers of deaths from all causes, as well as from various cause-specific UCODs. In the calculation of SMR, we counted the observed person-years for each study participant from the date of enrollment in the cohort to either the date of death or the final day of 2016. We determined the expected number of deaths for the dementia cohort using the personyear approach. This involved using the age-specific (in 10-year intervals) and sex-specific annual mortality rates of all causes and various specific UCODs, with reference to the general population in Taiwan between 2005 and 2016. Age at enrollment was categorized into 35-49 years, 50-64 years, 65-79 years, and ≥ 80 years. During the follow-up period, the study cohort contributed 128,142 person-years. We calculated the expected numbers of deaths, with reference to the age-sex-calendar-specific mortality rates of all causes and various specific UCODs in the general population. The analysis was performed using SAS version 9.4 (SAS Institute Inc., Cary, NC, USA), and the level of significance was set at α= 0.05.

### Ethics statement

Data collection and analysis were performed in accordance with the guidelines set forth by the Declaration of Helsinki. The study proposal was approved by the Institutional Review Board of Tsaotun Psychiatric Center, of the Ministry of Health and Welfare (IRB No. 109004). All data management and analyses were conducted at the HWDSC, of the Ministry of Health and Welfare, to ensure data security and confidentiality.

## RESULTS

The study cohort included 33,680 dementia cases, with a slight female predominance. The mean± standard deviation age at the time of cohort enrollment was 75.21± 9.25 years (median, 77). The mean duration of follow-up was 3.85± 2.93 years. A total of 13,485 all-cause deaths were noted between 2005 and 2016. Among the deceased individuals, the mean ± standard deviation age at death was 82.00± 8.33 years, with most deaths (n= 13,029, 96.6%) occurring in individuals aged 65 years and older (Table 1).

### Standardized mortality ratios associated with dementia

Circulatory diseases emerged as the leading UCOD (n= 3,505), accounting for 26.0% of the total deaths. This was followed by respiratory system diseases (n = 2,875, 21.3%); cancer (n = 1,724, 12.8%); and endocrine, nutritional, and metabolic diseases (n= 1,083, 8.0%; Table 2).

Compared to the general population, PWD were found to have a significantly higher risk of all-cause mortality (SMR, 2.01; 95% confidence interval [CI], 1.97 to 2.04). A cause-specific analysis revealed that the highest SMR was observed for diseases of the nervous system (SMR, 7.58; 95% CI, 7.02 to 8.17), followed by mental, behavioral, and neurodevelopmental disorders (SMR, 4.80; 95% CI, 4.28 to 5.36). Significantly elevated SMRs were also noted for skin diseases (SMR, 2.69; 95% CI, 2.17 to 3.30) and respiratory system diseases (SMR, 2.57; 95% CI, 2.48 to 2.66), including pneumonia (SMR, 2.89; 95% CI, 2.76 to 3.04). Other conditions such as infections (SMR, 2.33; 95% CI, 2.14 to 2.52); endocrine, nutritional, and metabolic diseases (SMR, 2.25; 95% CI, 2.12 to 2.39); genitourinary system diseases (SMR, 2.19; 95% CI, 2.05 to 2.34), circulatory diseases (SMR, 1.98; 95% CI, 1.91 to 2.05), and diseases of the musculoskeletal system and connective tissue (SMR, 1.73; 95% CI, 1.42 to 2.09) also exhibited increased SMRs. In addition, dementia was associated with significantly elevated SMRs for accidents (SMR, 1.27; 95% CI, 1.09 to 1.47) and suicide (SMR, 1.82; 95% CI, 1.44 to 2.27) (Table 2). More detailed cause-specific SMRs are shown in Supplementary Material 1.

### Standardized mortality ratios by sex

Table 2 also presents sex-specific SMRs. Among both females and males, dementia was significantly associated with elevated all-cause mortality, with SMRs of 1.92 (95% CI, 1.87 to 1.97) and 2.09 (95% CI, 2.04 to 2.13), respectively. For female, the highest cause-specific SMR was associated with nervous system diseases (SMR, 6.76), followed by mental and behavioral disorders (SMR, 4.51) and skin and subcutaneous tissue diseases (SMR, 3.12). For male, nervous system diseases (SMR, 8.35) and mental and behavioral disorders (SMR, 5.24) were similarly the UCODs with the most elevated SMRs. Supplementary Material 1 provides a more detailed breakdown of cause-specific SMRs for both males and females.

### Standardized mortality ratios by age

Table 3 presents both overall and cause-specific SMRs, stratified by age. Although the all-cause SMRs were significantly elevated across all age groups, the SMR decreased with age. The highest SMR was observed in patients who were enrolled between the ages of 35-49 years (SMR, 8.52), followed by those who enrolled at 50-64 years (SMR, 5.29), 65-79 years (SMR, 2.55), and ≥ 80 years (SMR, 1.79). The estimates of cause-specific SMRs were particularly reliable for younger patients. Notable increases in SMR were observed for nervous system diseases (SMRs, 12.05 and 5.69, respectively) and mental disorders (SMRs, 10.87 and 4.08, respectively) in older patients aged 65-79 years and ≥ 80 years. Additionally, elevated SMRs were noted for respiratory diseases (SMR, 4.04) and skin and subcutaneous tissue diseases (SMR, 4.53) in patients aged 65-79 years.

## DISCUSSION

This was the first study to examine both overall and cause-specific mortality rates among PWD in Taiwan. Our research revealed that PWD had approximately double the risk of all-cause mortality relative to those without dementia. While differences in statistical methods and the presentation of results (for example, the use of SMRs and age-specific mortality rates) may hinder direct comparisons with previous studies, the overall SMR for dementia in our study was 2.01. This is comparable to studies conducted in the United States and Finland, but lower than ratios found in some Eastern European countries [12-15]. Our research suggests that the younger the patient at cohort enrollment, the higher the SMRs for causes associated with dementia. Few population-based studies have reported age-specific and sex-specific mortality risks related to dementia. One study in Finland similarly indicated that SMRs decreased with increasing age. In that study, relative to the general population, the risk of death was approximately 3 times higher among individuals with AD who were younger than 80 years, twice as high among those aged 80 years to 89 years, and 1.5 times higher among individuals aged 90 years and older [13]. The decrease in SMR with increasing age could be attributed to the diminished impact of dementia with age. However, further analysis of the UCODs in younger PWD in our study, as discussed below, allowed us to better understand the risk of death in these age groups.

The distribution of UCODs for dementia in Taiwan mirrors that of other countries. In our study, circulatory diseases emerged as the leading UCOD, accounting for 25.99% of total deaths. This was followed by diseases of the respiratory system, which accounted for 21.32%. In a cohort study based on the Swedish Dementia Registry, the most frequent UCOD was cardiovascular disease, at 37% [5]. Another study, which examined autopsy reports, concluded that the 2 most common causes of death in PWD were bronchopneumonia (38.4%) and ischemic heart disease (23.1%) [16]. The greatest SMR was observed for nervous system diseases (SMR, 7.58). Cerebrovascular disease is a major cause of dementia, and both conditions share numerous common etiologies and several vascular risk factors. The high SMR for nervous system diseases in dementia underscores the close correlation between these diseases.

Many mental disorders, such as depression or schizophrenia, are risk factors for dementia [17,18]. Additionally, the behavioral and psychological symptoms of dementia are frequently classified as corresponding mental disorders in health insurance claims. This could account for the high SMR observed for mental, behavioral, and neurodevelopmental disorders in our study (SMR, 4.80). This may also partially explain why dementia was associated with significantly elevated SMRs for violence and accidents (SMR, 1.27) as well as suicide (SMR, 1.82), which are related to mental disorders. Conversely, other studies have reported dementia as the primary registered underlying cause of death in individuals diagnosed with the condition, reflecting a growing awareness of the potential mortality risk associated with dementia [19].

An elevated SMR was observed for diseases of the respiratory system (SMR, 2.57), including pneumonia (SMR, 2.89) and infection (SMR, 2.33). Dementia was found to be associated with an increased risk of death from pneumonia [20]. A systematic review of 13 relevant studies in a meta-analysis revealed that the odds of death from pneumonia were significantly higher for individuals with any form of dementia compared to those without dementia (odds ratio, 2.22; 95% CI, 1.44 to 3.42; p< 0.001) [21]. The risk of upper respiratory infection progressing to pneumonia was also increased due to diminished cognitive function and self-care abilities in PWD. Medications have been found to influence pneumonia among PWD. Specifically, dementia has been associated with higher short-term mortality following pneumonia, particularly in users of antipsychotics [22]. Other medications, such as antidementia drugs, antiepileptic drugs, and anticholinergic drugs, have been reported to increase the risk of pneumonia in PWD [23-25]. In the present study, endocrine, nutritional, and metabolic diseases also showed a moderately increased SMR (2.25), with diabetes accounting for 93% of person-years. Diabetes mellitus is not only a risk factor for dementia, but is also associated with the risk of mortality in PWD [26-29].

Our study revealed a distinct inverse relationship between the age at which individuals were enrolled in the cohort and both allcause and nearly every cause-specific SMR. As age increases, the mortality rate from various causes in the general population also increases, thereby reducing the dominance of dementia as a determinant of mortality. In the age groups of 35-49 years and 50-64 years, the largest increases in SMR were observed for nervous system diseases (SMR, 61.75 and 48.91, respectively), respiratory system diseases (SMR, 23.11 and 13.19, respectively), and circulatory diseases (SMR, 14.71 and 8.70, respectively). Early-onset dementia, which occurs in individuals younger than 65 years, merits particular attention, as this age group is typically engaged in labor and productivity when the disease manifests. Compared to lateonset dementia, the early-onset dementia group exhibited fewer AD cases but more cases attributed to vascular issues, traumatic brain injury, alcohol, and the human immunodeficiency virus [30]. Given that early-onset dementia is strongly associated with numerous neurological and vascular factors, the mortality rates from these diseases are most pronounced in this age group. Effective management of vascular risk factors in this age group, such as controlling hypertension and hyperlipidemia, is crucial for slowing the progression of dementia and reducing mortality rates. In contrast, pneumonia, an acute respiratory infection with diverse causes, is relatively uncommon in younger age groups. The high SMR of pneumonia in early-onset dementia may be linked to an impaired gag reflex and decreased immunity, which are linked to early-onset dementia mentioned above. Consequently, patients with early-onset dementia require careful management and prevention of pneumonia to maximize life expectancy and quality of life.

To ensure the accuracy of diagnosis, we included only individuals who had a primary diagnosis of dementia, supported by at least 2 outpatient medical claims or 1 hospital admission within a 1-year period. This aligns with criteria frequently used in previous Taiwanese studies that similarly utilized NHI claim data to identify dementia cases [29,31]. Our study sample consisted of 29,705 patients with dementia aged 65 years and older, which represented a prevalence of 1.3% among the elderly population in 2005 (approximately 10% of the elderly population that year). Fuh & Wang [32] reported that community surveys conducted in Taiwan indicated a dementia prevalence of approximately 1.7-4.3% in 2007 among the elderly, suggesting that some older individuals with dementia in the community were not receiving treatment.

This study had certain limitations. First, the potential misclassification of UCODs on death certificates could result in inaccurate SMRs. While the data quality for the TDR has been evaluated and deemed valid [11], autopsies are not commonly performed in Taiwan due to cultural considerations. Some studies have suggested that autopsies reveal a higher rate of pneumonia-related mortality among PWD, which could indicate an underestimation of respiratory system diseases [33]. Second, the recent updates to long-term care policy in Taiwan may have improved the prognosis of dementia. However, this study does not provide a secular trend of cause-specific mortality, primarily due to an inadequate sample size. Third, while we examined the overall and cause-specific mortality in PWD, we did not analyze the prevalence data of dementia-related diseases. We relied on mortality data rather than incidence or prevalence data, resulting in a conflation of risk and prognosis concepts. Consequently, the potential for further causal inference between dementia and other diseases may be limited. Finally, the covariates related to mortality, such as cognition level or frailty, were not available from the datasets analyzed in this study. Therefore, their contributions to cause-specific mortality could not be analyzed. Despite these limitations, this study does possess certain methodological strengths. These include its population-based design and an analysis of cause-specific mortality in relation to dementia. This provides valuable insights for developing care strategies aimed at reducing mortality from specific causes.

## Figures and Tables

**Figure f1-epih-45-e2023082:**
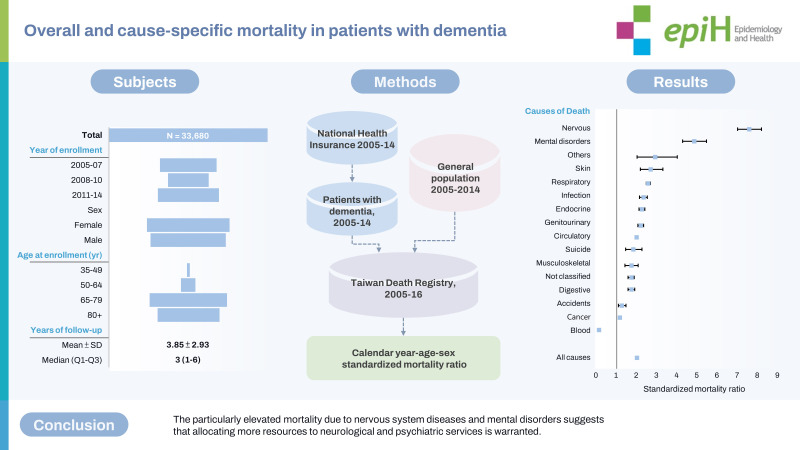


**Table 1.** Characteristics of study participants

Characteristics	n (%)
Total	33,680 (100)
Calendar year of enrollment^1^	
2005-2007	11,952 (35.5)
2008-2010	8,684 (25.8)
2011-2014	13,044 (38.7)
Sex	
Female	17,703 (52.6)
Male	15,977 (47.4)
Age at cohort enrollment (yr)	
35-49	748 (2.2)
50-64	3,227 (9.6)
65-79	16,571 (49.2)
≥80	13,134 (39.0)
Mean±SD	75.21±9.25
Median (Q1-Q3)	77 (70-83)
Years of follow-up	
Mean±SD	3.85±2.93
Median (Q1-Q3)	3 (1-6)
Survival status by the end of 2016	
Survivors	20,195 (60.0)
Non-survivors	13,485 (40.0)
Age at death (yr)	
35-49	65 (0.5)
50-64	391 (2.9)
65-79	3,919 (29.1)
≥80	9,110 (67.6)
Mean±SD	82.00±8.33
Median (Q1-Q3)	83 (78-88)

SD, standard deviation; Q, quartile.

1 The year of the study participant’s first clinical visit between 2005 and 2014.

**Table 2.** Overall and sex-specific SMRs for all causes and major specific causes in persons with dementia

Causes of death		Total (PY=128,142)			Female (PY=69,707)			Male (PY=58,435)		
		No. of deaths		SMR (95% CI)	No. of deaths		SMR (95% CI)	No. of deaths		SMR (95% CI)
		Exp.	Obs.		Exp.	Obs.		Exp.	Obs.	
All causes		6,722	13,485	2.01 (1.97, 2.04)	3,257	6,257	1.92 (1.87, 1.97)	3,465	7,228	2.09 (2.04, 2.13)
	Infection	254.13	591	2.33 (2.14, 2.52)	117.53	252	2.14 (1.89, 2.43)	136.60	339	2.48 (2.22, 2.76)
	Cancer	1,465	1,724	1.18 (1.12, 1.23)	611.97	735	1.20 (1.12, 1.29)	852.97	989	1.16 (1.09, 1.23)
	Endocrine, nutritional, and metabolic diseases	481.80	1,083	2.25 (2.12, 2.39)	293.00	647	2.21 (2.04, 2.39)	188.80	436	2.31 (2.10, 2.54)
	Blood and blood-forming organs	403.75	52	0.13 (0.10, 0.17)	246.73	26	0.11 (0.07, 0.15)	157.02	26	0.17 (0.11, 0.24)
	Mental and behavioral disorders	65.44	314	4.80 (4.28, 5.36)	39.48	178	4.51 (3.87, 5.22)	25.96	136	5.24 (4.40, 6.20)
	Nervous system	91.17	691	7.58 (7.02, 8.17)	43.96	297	6.76 (6.01, 7.57)	47.21	394	8.35 (7.54, 9.21)
	Circulatory system	1,771	3,505	1.98 (1.91, 2.05)	930.10	1,688	1.81 (1.73, 1.90)	840.65	1,817	2.16 (2.06, 2.26)
	Respiratory system	1,119	2,875	2.57 (2.48, 2.66)	439.67	1,075	2.45 (2.30, 2.60)	679.42	1,800	2.65 (2.53, 2.77)
	Digestive system	336.64	575	1.71 (1.57, 1.85)	173.46	285	1.64 (1.46, 1.85)	163.18	290	1.78 (1.58, 1.99)
	Genitourinary system	411.83	901	2.19 (2.05, 2.34)	241.80	486	2.01 (1.84, 2.20)	170.03	415	2.44 (2.21, 2.69)
	Skin and subcutaneous tissue	34.56	93	2.69 (2.17, 3.30)	19.89	62	3.12 (2.39, 4.00)	14.67	31	2.11 (1.44, 3.00)
	Musculoskeletal system and connective tissue	62.86	109	1.73 (1.42, 2.09)	36.64	53	1.45 (1.08, 1.89)	26.22	56	2.14 (1.61, 2.77)
	Symptoms not elsewhere classified	393.93	677	1.72 (1.59, 1.85)	212.47	355	1.67 (1.50, 1.85)	181.46	322	1.77 (1.59, 1.98)
	Accidents	141.81	180	1.27 (1.09, 1.47)	55.71	67	1.20 (0.93, 1.53)	86.10	113	1.31 (1.08, 1.58)
	Suicide	43.30	79	1.82 (1.44, 2.27)	16.21	34	2.10 (1.45, 2.93)	27.09	45	1.66 (1.21, 2.22)
	Others	12.46	36	2.89 (2.02, 4.00)	6.01	17	2.83 (1.65, 4.53)	6.45	19	2.94 (1.77, 4.60)

SMRs, standardized mortality ratios; PY, person-years; Exp., expected; Obs., observed; CI, confidence interval.

**Table 3.** Age-specific SMRs for all causes and major specific causes in persons with dementia

Causes of death		Age (yr)											
		35-49 (PY=2,967)			50-64 (PY=11,132)			65-79 (PY=58,894)			≥80 (PY=55,149)		
		No. of deaths		SMR (95% CI)	No. of deaths		SMR (95% CI)	No. of deaths		SMR (95% CI)	No. of deaths		SMR (95% CI)
		Exp.	Obs.		Exp.	Obs.		Exp.	Obs.		Exp.	Obs.	
All causes		7.63	65	8.52 (6.57, 10.86)	73.14	387	5.29 (4.78, 5.85)	1,525	3,888	2.55 (2.47, 2.63)	5,117	9,145	1.79 (1.75, 1.82)
	Infection	0.18	1	5.69 (0.07, 31.63)	1.63	11	6.75 (3.37, 12.08)	47.67	160	3.36 (2.86, 3.92)	204.70	419	2.05 (1.86, 2.25)
	Cancer	2.49	14	5.61 (3.07, 9.42)	31.23	74	2.37 (1.86, 2.97)	487.80	621	1.27 (1.17, 1.38)	943.40	1,015	1.08 (1.01, 1.14)
	Endocrine, nutritional, and metabolic diseases	0.25	3	11.87 (2.39, 34.67)	4.59	30	6.53 (4.41, 9.33)	132.30	429	3.24 (2.94, 3.57)	344.70	621	1.80 (1.66, 1.95)
	Blood and blood-forming organs	0.20	0	N/A	3.59	2	0.56 (0.06, 2.01)	103.10	8	0.08 (0.03, 0.15)	296.90	42	0.14 (0.10, 0.19)
	Mental and behavioral disorders	0.09	0	N/A	0.36	3	8.40 (1.69, 24.55)	6.72	73	10.87 (8.52, 13.66)	58.28	238	4.08 (3.58, 4.64)
	Nervous system	0.10	6	61.75 (22.55, 134.4)	0.80	39	48.91 (34.77, 66.86)	20.74	250	12.05 (10.61, 13.65)	69.54	396	5.69 (5.15, 6.28)
	Circulatory system	1.09	16	14.71 (8.41, 23.90)	12.98	113	8.70 (7.17, 10.46)	355.30	969	2.73 (2.56, 2.90)	1,401	2,407	1.72 (1.65, 1.79)
	Respiratory system	0.22	5	23.11 (7.45, 53.92)	3.18	42	13.19 (9.51, 17.83)	167.18	675	4.04 (3.74, 4.35)	948.50	2,153	2.27 (2.17, 2.37)
	Digestive system	1.01	9	8.90 (4.06, 16.89)	5.91	14	2.37 (1.29, 3.97)	87.52	173	1.98 (1.69, 2.29)	242.20	379	1.56 (1.41, 1.73)
	Genitourinary system	0.11	0	N/A	2.10	14	6.65 (3.63, 11.16)	79.24	226	2.85 (2.49, 3.25)	330.40	661	2.00 (1.85, 2.16)
	Skin and subcutaneous tissue	0.02	0	N/A	0.18	0	N/A	6.18	28	4.53 (3.01, 6.55)	28.17	65	2.31 (1.78, 2.94)
	Musculoskeletal system and connective tissue	0.06	0	N/A	0.52	2	3.82 (0.43, 13.80)	10.81	22	2.04 (1.28, 3.08)	51.48	85	1.65 (1.32, 2.04)
	Symptoms not elsewhere classified	0.37	2	5.43 (0.61, 19.61)	2.08	10	4.81 (2.30, 8.84)	45.72	131	2.87 (2.40, 3.40)	345.80	534	1.54 (1.42, 1.68)
	Accidents	0.79	5	6.35 (2.05, 14.82)	4.13	15	3.64 (2.03, 6.00)	47.80	69	1.44 (1.12, 1.83)	89.10	91	1.02 (0.82, 1.25)
	Suicide	0.67	3	4.49 (0.90, 13.12)	2.57	11	4.27 (2.13, 7.64)	17.92	44	2.45 (1.78, 3.30)	22.13	21	0.95 (0.59, 1.45)
	Others	0.10	1	9.69 (0.13, 53.89)	0.39	7	18.02 (7.22, 37.13)	3.98	10	2.51 (1.20, 4.62)	7.99	18	2.25 (1.34, 3.56)

SMRs, standardized mortality ratios; PY, person-years; Exp., expected; Obs., observed; CI, confidence interval; N/A, not available.

## SUPPLEMENTARY MATERIALS

Supplement Material 1. Overall and sex-specific standardized mortality ratio (SMR) of all causes and detailed specific causes in PWD
