# Supplementary material for: Overall and cause-specific mortality in patients with dementia: a population-based cohort study in Taiwan
Source: Epidemiol Health. 2023 Aug 31;45:e2023082. doi: 10.4178/epih.e2023082 (PMC10728617; doi:10.4178/epih.e2023082)
Supplement: Supplement Material 1. — Overall and sex-specific standardized mortality ratio (SMR) of all causes and detailed specific causes in PWD [file epih-45-e2023082-Supplementary-1.docx]

Supplementary Material 1. Overall and sex-specific standardized mortality ratio (SMR) of all causes and detailed specific causes in PWD

| Causes of death | Total (PY=128142) | | | | | Female (PY=69707) | | | | | Male (PY=58435) | | | | |
| --- | --- | --- | --- | --- | --- | --- | --- | --- | --- | --- | --- | --- | --- | --- | --- |
|  | No. of death | | SMR | 95% CI | | No. of death | | SMR | 95% CI | | No. of death | | SMR | 95% CI | |
|  | Exp. | Obs. |  |  |  | Exp. | Obs. |  |  |  | Exp. | Obs. |  |  |  |
| All causes | 6722 | 13485 | 2.01 | 1.97- | 2.04 | 3257 | 6257 | 1.92 | 1.87- | 1.97 | 3,465 | 7228 | 2.09 | 2.04- | 2.13 |
| Infection | 254.13 | 591 | 2.33 | 2.14- | 2.52 | 117.53 | 252 | 2.14 | 1.89- | 2.43 | 136.60 | 339 | 2.48 | 2.22- | 2.76 |
| Neoplasm | 1465 | 1724 | 1.18 | 1.12- | 1.23 | 611.97 | 735 | 1.20 | 1.12- | 1.29 | 852.97 | 989 | 1.16 | 1.09- | 1.23 |
| Oral | 44.00 | 52 | 1.18 | 0.88- | 1.55 | 9.35 | 8 | 0.86 | 0.37- | 1.69 | 34.65 | 44 | 1.27 | 0.92- | 1.70 |
| Breast (females only) | 28.67 | 35 | 1.22 | 0.85- | 1.70 | 28.24 | 35 | 1.24 | 0.86- | 1.72 | 0.43 | 0 | a | a | a |
| Cervix uteri (females only) | 21.94 | 35 | 1.60 | 1.11- | 2.22 | 21.94 | 35 | 1.60 | 1.11- | 2.22 | 0.00 | 0 | a | a | a |
| Ovary (females only) | 9.29 | 12 | 1.29 | 0.67- | 2.26 | 9.29 | 12 | 1.29 | 0.67- | 2.26 | 0.00 | 0 | a | a | a |
| Prostate (males only) | 63.51 | 77 | 1.21 | 0.96- | 1.52 | 0.00 | 0 | a | a | a | 63.51 | 77 | 1.21 | 0.96- | 1.52 |
| Kidney | 24.07 | 29 | 1.20 | 0.81- | 1.73 | 12.35 | 20 | 1.62 | 0.99- | 2.50 | 11.72 | 9 | 0.77 | 0.35- | 1.46 |
| Stomach | 99.19 | 122 | 1.23 | 1.02- | 1.47 | 36.54 | 41 | 1.12 | 0.81- | 1.52 | 62.65 | 81 | 1.29 | 1.03- | 1.61 |
| Colon rectum and anus | 205.59 | 268 | 1.30 | 1.15- | 1.47 | 96.87 | 134 | 1.38 | 1.16- | 1.64 | 108.72 | 134 | 1.23 | 1.03- | 1.46 |
| Pancreas | 56.82 | 55 | 0.97 | 0.73- | 1.26 | 28.51 | 28 | 0.98 | 0.65- | 1.42 | 28.31 | 27 | 0.95 | 0.63- | 1.39 |
| Liver and intrahepatic bile ducts | 236.30 | 281 | 1.19 | 1.05- | 1.34 | 102.50 | 125 | 1.22 | 1.02- | 1.45 | 133.79 | 156 | 1.17 | 0.99- | 1.36 |
| Bronchus and lung | 322.12 | 331 | 1.03 | 0.92- | 1.14 | 111.89 | 130 | 1.16 | 0.97- | 1.38 | 210.23 | 201 | 0.96 | 0.83- | 1.10 |
| Others | 303.65 | 427 | 1.41 | 1.28- | 1.55 | 129.41 | 167 | 1.29 | 1.10- | 1.50 | 174.24 | 260 | 1.49 | 1.32- | 1.68 |
| Endocrine nutritional and metabolic diseases | 481.80 | 1083 | 2.25 | 2.12- | 2.39 | 293.00 | 647 | 2.21 | 2.04- | 2.39 | 188.80 | 436 | 2.31 | 2.10- | 2.54 |
| Diabetes | 447.97 | 1007 | 2.25 | 2.11- | 2.39 | 273.65 | 601 | 2.20 | 2.02- | 2.38 | 174.32 | 406 | 2.33 | 2.11- | 2.57 |
| Others | 12.81 | 76 | 5.93 | 4.67- | 7.43 | 7.37 | 46 | 6.24 | 4.57- | 8.32 | 5.44 | 30  Continued | 5.52 | 3.72- | 7.88 |
| Blood and blood- forming organs | 403.75 | 52 | 0.13 | 0.10- | 0.17 | 246.73 | 26 | 0.11 | 0.07- | 0.15 | 157.02 | 26 | 0.17 | 0.11- | 0.24 |
| Mental and behavioral disorders | 65.44 | 314 | 4.80 | 4.28- | 5.36 | 39.48 | 178 | 4.51 | 3.87- | 5.22 | 25.96 | 136 | 5.24 | 4.40- | 6.20 |
| Nervous System | 91.17 | 691 | 7.58 | 7.02- | 8.17 | 43.96 | 297 | 6.76 | 6.01- | 7.57 | 47.21 | 394 | 8.35 | 7.54- | 9.21 |
| Circulatory system | 1771 | 3505 | 1.98 | 1.91- | 2.05 | 930.10 | 1688 | 1.81 | 1.73- | 1.90 | 840.65 | 1817 | 2.16 | 2.06- | 2.26 |
| Heart disease | 945.96 | 1714 | 1.81 | 1.73- | 1.90 | 505.34 | 863 | 1.71 | 1.60- | 1.83 | 440.62 | 851 | 1.93 | 1.80- | 2.07 |
| Cerebral vascular | 593.00 | 1495 | 2.52 | 2.39- | 2.65 | 303.68 | 654 | 2.15 | 1.99- | 2.33 | 289.32 | 841 | 2.91 | 2.71- | 3.11 |
| Hypertension | 125.13 | 219 | 1.75 | 1.53- | 2.00 | 72.40 | 129 | 1.78 | 1.49- | 2.12 | 52.73 | 90 | 1.71 | 1.37- | 2.10 |
| Others | 50.47 | 77 | 1.53 | 1.20- | 1.91 | 22.99 | 42 | 1.83 | 1.32- | 2.47 | 27.48 | 35 | 1.27 | 0.89- | 1.77 |
| Respiratory system | 1119 | 2875 | 2.57 | 2.48- | 2.66 | 439.67 | 1075 | 2.45 | 2.30- | 2.60 | 679.42 | 1800 | 2.65  Continued | 2.53- | 2.77 |
| Pneumonia | 587.93 | 1702 | 2.89 | 2.76- | 3.04 | 258.78 | 678 | 2.62 | 2.43- | 2.82 | 329.15 | 1024 | 3.11 | 2.92- | 3.31 |
| Chronic obstructive pulmonary disease | 380.62 | 846 | 2.22 | 2.08- | 2.38 | 115.81 | 261 | 2.25 | 1.99- | 2.54 | 264.81 | 585 | 2.21 | 2.03- | 2.40 |
| Pneumoconiosis | 46.70 | 114 | 2.44 | 2.01- | 2.93 | 17.18 | 45 | 2.62 | 1.91- | 3.50 | 29.51 | 69 | 2.34 | 1.82- | 2.96 |
| Others | 103.82 | 213 | 2.05 | 1.79- | 2.35 | 47.89 | 91 | 1.90 | 1.53- | 2.33 | 55.93 | 122 | 2.18 | 1.81- | 2.60 |
| Digestive system | 336.64 | 575 | 1.71 | 1.57- | 1.85 | 173.46 | 285 | 1.64 | 1.46- | 1.85 | 163.18 | 290 | 1.78 | 1.58- | 1.99 |
| Genitourinary system | 411.83 | 901 | 2.19 | 2.05- | 2.34 | 241.80 | 486 | 2.01 | 1.84- | 2.20 | 170.03 | 415 | 2.44 | 2.21- | 2.69 |
| Skin and subcutaneous tissue | 34.56 | 93 | 2.69 | 2.17- | 3.30 | 19.89 | 62 | 3.12 | 2.39- | 4.00 | 14.67 | 31 | 2.11 | 1.44- | 3.00 |
| Musculoskeletal system and connective tissue | 62.86 | 109 | 1.73 | 1.42- | 2.09 | 36.64 | 53 | 1.45 | 1.08- | 1.89 | 26.22 | 56 | 2.14  Continued | 1.61- | 2.77 |
| Symptoms not elsewhere classified | 393.93 | 677 | 1.72 | 1.59- | 1.85 | 212.47 | 355 | 1.67 | 1.50- | 1.85 | 181.46 | 322 | 1.77 | 1.59- | 1.98 |
| Accidents | 141.81 | 180 | 1.27 | 1.09- | 1.47 | 55.71 | 67 | 1.20 | 0.93- | 1.53 | 86.10 | 113 | 1.31 | 1.08- | 1.58 |
| Suicide | 43.30 | 79 | 1.82 | 1.44- | 2.27 | 16.21 | 34 | 2.10 | 1.45- | 2.93 | 27.09 | 45 | 1.66 | 1.21- | 2.22 |
| Others | 12.46 | 36 | 2.89 | 2.02- | 4.00 | 6.01 | 17 | 2.83 | 1.65- | 4.53 | 6.45 | 19 | 2.94 | 1.77- | 4.60 |

a: Not calculable

CI: confidence interval; PY: person-years
